# Maternal Health and Sociodemographic Characteristics Influence Infant Growth to 24 Months in the Tunza Mwana Cohort: A Prospective Cohort Study

**DOI:** 10.1111/mcn.70195

**Published:** 2026-05-14

**Authors:** David Taylor Hendrixson, Ruchi Tiwari, Philip James, Zulfiqar A. Bhutta, André Briend, Sheila Isanaka, Tanya Khara, Natasha Lelijveld, Mark J. Manary, Andrew Mertens, Sophie E. Moore, Kieran S. O'Brien, Jonathan Wells, Priscah Lihanda, Eric Ochola, Grace Aldrovandi, Benson O. Singa, Christine J. McGrath

**Affiliations:** ^1^ Department of Pediatrics University of Washington Seattle Washington USA; ^2^ Department of Global Health University of Washington Seattle Washington USA; ^3^ Emergency Nutrition Network Oxford UK; ^4^ Center for Global Child Health Hospital for Sick Children Toronto Canada; ^5^ Institute for Global Health & Development The Aga Khan University Karachi Pakistan; ^6^ Center for Child Health Research Tampere University Tampere Finland; ^7^ Department of Nutrition, Exercise and Sports University of Copenhagen Copenhagen Denmark; ^8^ Harvard T.H. Chan School of Public Health Boston Massachusetts USA; ^9^ Epicentre Paris France; ^10^ Department of Pediatrics Washington University in St. Louis St. Louis Missouri USA; ^11^ School of Public Health University of California Berkeley Berkeley California USA; ^12^ Kings College London London UK; ^13^ Medical Research Unit the Gambia at the London School of Hygiene and Tropical Medicine Fajara The Gambia; ^14^ Francis I. Proctor Foundation University of California San Francisco California USA; ^15^ Great Ormond Street Institute of Child Health (ICH) University College London (UCL) London UK; ^16^ Kenya Medical Research Institute Nairobi Kenya; ^17^ Department of Pediatrics University of California Los Angeles Los Angeles California USA

**Keywords:** infant growth, Kenya, maternal health, stunting, underweight, wasting

## Abstract

Upstream pathways influencing child growth are complex. Weight‐for‐age z‐score (WAZ) reflects both ponderal and linear growth and can identify children at high mortality risk. Using data from a prospective cohort of 326 mother‐child pairs in Kenya, we evaluated whether associations between maternal exposures and child growth outcomes in early and later childhood. Growth trajectories were examined using locally weighted scatterplot smoothing and piecewise linear mixed‐effect models with a knot at age 3 months. Poisson regression models examined associations between maternal characteristics and child underweight (WAZ < −2) and stunting (LAZ < −2) before and after 3 months. Mean WAZ increased to a peak at 3 months before declining steadily through 24 months. Lower maternal education (adjusted β [aβ] −0.18; 95% CI: −0.24, −0.13), household crowding (aβ −0.2; 95% CI: −0.28, −0.11), lower wealth quintile (aβ −0.04; 95% CI: −0.06, −0.02) and multiparity (aβ −0.16; 95% CI: −0.23, −0.1) were associated with lower monthly rate of change in WAZ prior to age 3 months. Younger maternal age was associated with an increased monthly rate of change in WAZ (aβ = 0.13; 95% CI: 0.08, 0.18) before 3 months and decreased monthly WAZ (aβ −0.02; 95% CI: −0.03, −0.01) after 3 months. Preterm birth was associated with increased risk of underweight in both periods (aRR 2.77; 95% CI: 1.39–5.50). Maternal mental health, intimate partner violence and adverse childhood experiences were not significantly associated with child growth. Maternal nutritional, socio‐economic and household‐level factors shape early growth, emphasising the need for cross‐sectoral programs to support growth.

## Summary

- Upstream factors beyond nutrition that influence child growth are complex and not well understood.
- Maternal nutritional status, education and socioeconomic status were associated with child growth outcomes to 24 months.
- Factors associated with child growth differed in early infancy (< 3 months) and later infancy (> 3 months), underscoring the importance of timely programs to impact child growth.
- These findings highlight the need for cross‐sectoral collaborations to develop innovative programs that address the diverse factors associated with child growth in the first 2 years of life.

**Abbreviations**
ACE‐Q: adverse childhood experiences questionnaire
ART: antiretroviral therapy
GAD‐7: generalised anxiety disorder 7‐item
MDD‐W: minimum dietary diversity for women
MUAC: mid‐upper arm circumference
PHQ‐9: patient health questionnaire‐9
WLWH: women living with HIV

## 1 Introduction

Childhood malnutrition remains a significant problem globally (United Nations Children's Fund, World Health Organization, International Bank for Reconstruction and Development/The World Bank 2025). Historically, there has been a focus on wasting (low weight‐for‐length *z*‐score) and stunting (low length‐for‐age *z*‐score); however, emerging evidence suggests that these measures alone may not effectively identify children at the highest risk of death and, when used in isolation, they miss the overlap between wasting and stunting (Myatt et al. 2018). Furthermore, studies have shown that weight‐for‐length can be difficult to measure accurately in resource‐constrained environments (Mwangome and Berkley 2014). Given this, focus has shifted to the weight‐for‐age *z*‐score (WAZ), which reflects both ponderal and linear growth (Sadler et al. 2021). Low WAZ (< −3) identifies children with concurrent wasting and stunting (Myatt et al. 2018) and is strongly associated with elevated mortality risk (Khara et al. 2023).

The upstream pathways leading to underweight, wasting, stunting and concurrent wasting and stunting are complex and are not clearly understood, yet they represent important areas for nutrition policy and program development (Sadler et al. 2022; Wells et al. 2019). These upstream factors interact to constrain child growth and development. Comprehensive characterisation of maternal risk factors underlying infant and child growth over time may help to identify key risk factors driving these interconnected processes and elucidate conditions amenable to public health and clinical interventions to prevent various forms of undernutrition. Considerable evidence indicates that the risk factors and causes of underweight, wasting and stunting often overlap (Mertens et al. 2023), presenting an opportunity for integrated programming to address these conditions together. By focusing on shared pathways to growth faltering, public health and clinical interventions may be aligned to enhance efficiency and maximise impact across multiple forms of undernutrition.

Maternal and household characteristics have been associated with childhood growth outcomes in large‐scale studies. A cross‐sectional analysis of data from 35 low‐ and middle‐income countries (LMICs) identified low maternal height, lower maternal body mass index (BMI), low maternal education and low socioeconomic status as significant risk factors for childhood underweight, stunting and wasting (Li et al. 2020a). Similar risk factors have been identified for infant underweight and wasting across survey data from 56 LMICs (Kerac et al. 2025). Additionally, several large‐scale studies have demonstrated an association between concurrent wasting and stunting and factors such as the lowest socioeconomic status, lack of maternal formal education and maternal underweight (Amadu et al. 2021; Chowdhury et al. 2022). While previous studies have examined associations between maternal factors and child growth outcomes, most have focused primarily on maternal anthropometric, health and socioeconomic characteristics (Mertens et al. 2023). These studies have often lacked assessment of other potentially important factors, such as maternal mental health, adverse childhood experiences and exposure to intimate partner violence. Moreover, longitudinal data beginning in pregnancy provides a unique opportunity to identify upstream determinants of child growth trajectories that may be amenable to intervention.

We aimed to explore maternal factors as key upstream determinants of child growth trajectories from birth to 24 months of age, using data from a prospective longitudinal cohort. Our primary objective was to examine associations between maternal characteristics during late pregnancy and the exclusive breastfeeding period with child WAZ from birth to 24 months and with underweight (WAZ < −2) in the first 24 months, and whether associations between maternal exposures and child growth outcomes remained consistent or varied across different stages of early infancy. Secondary objectives included examining associations between maternal factors and length‐for‐age *z*‐score (LAZ) and weight‐for‐length *z*‐score (WLZ) over the same period, as well as stunting (LAZ < −2), and wasting (WLZ < −2) to 24 months. By identifying maternal factors that are consistently associated with child growth, and those that were specific to particular outcomes or time periods, we sought to inform the development of more targeted and timely prevention programs.

## 2 Methods

### 2.1 Study Setting and Population

The Tunza Mwana prospective cohort study enrolled pregnant women living with and without HIV at the Migori County Referral Hospital and St. Joseph's Mission Hospital in Migori County, Kenya. Women and their children were followed to 2‐years postpartum. The study was approved by the appropriate institutional review boards. All women provided written informed consent for themselves and their child to participate in the study.

Women were eligible for enrolment if they were between 28 and 42 weeks' gestation, aged 18–40 years, planning to primarily breastfeed their infant for at least 6 months, willing to utilise HIV services if living with HIV, and willing to provide written informed consent. The date of last menstrual period (LMP) was used to estimate the gestational age of pregnancy.

### 2.2 Outcomes

WAZ and underweight (WAZ < −2) were selected as the primary outcomes. These measures were chosen because they reflect both ponderal and linear growth (Sadler et al. 2021), underweight is strongly associated with mortality risk (Khara et al. 2023) and infant weight is generally measured more accurately than length. Secondary outcomes included LAZ, WLZ, stunting (LAZ < −2) and wasting (WLZ < −2).

### 2.3 Exposures

We examined associations with exposures in late pregnancy and the exclusive breastfeeding period (Supporting Information: Table S1). These exposures included maternal socio‐demographic characteristics (education, household wealth, crowding), clinical factors (anthropometry and illness), complicated pregnancy (infection, bleeding, high blood pressure, preeclampsia or COVID during pregnancy or hospitalised during pregnancy), prematurity, parity, dietary indicators (Minimum Dietary Diversity for Women [MDD‐W] (FAO and FHI 360 2016) and Household Food Insecurity Access Scale [HFIAS] (Jennifer Coates et al. 2007), anaemia in pregnancy, iron‐folic acid supplementation, mental health measures (patient health questionnaire‐9 [PHQ‐9], generalised anxiety disorder 7‐item [GAD‐7]), intimate partner violence (Soeken et al. 1998) and adverse childhood experiences (World Health Organization 2018).

### 2.4 Data Collection

Trained study staff collected data at enrolment and follow‐up visits within 7 days of delivery, at Weeks 3 and 6, and at months 3, 6, 9, 12, 18 and 24 postpartum. Women were interviewed at enrolment to collect sociodemographic and economic information, household food insecurity using the HFIAS, dietary diversity using the MDD‐W, depressive symptoms using the PHQ‐9 (Kroenke et al. 2001), anxiety symptoms using the GAD‐7 (Spitzer et al. 2006), intimate partner violence using the Abuse Assessment Screen (AAS) (Soeken et al. 1998), adverse childhood experiences by the Adverse Childhood Experiences Questionnaire (ACE‐Q) (World Health Organization 2018), current illness, and medical and obstetric history. Among women living with HIV (WLWH), data were collected on antiretroviral therapy (ART) regimen and other HIV‐related characteristics. Maternal height was measured at enrolment, and weight and mid‐upper arm circumference (MUAC) were measured at each visit.

At each postpartum visit, child data collection included anthropometric measurements (weight, length, head circumference and MUAC) obtained in triplicate using standard protocols, current health, history of recent illness and hospitalisations and medication use including antibiotics. Among children born to WLWH, data were collected on antiretroviral (ARV) prophylaxis from birth and receipt of cotrimoxazole, which begins at age 6 weeks per Kenya guidelines. Staff administered a standardised questionnaire to assess breastfeeding practices and introduction of other infant foods, including frequency of breastfeeding, 24‐h dietary intake in the mother and infant, and minimum dietary diversity (MDD) for children aged 6–23 months (Tufts University 2023). Infants born to WLWH underwent HIV PCR testing at week 6 and months 6 and 12 and HIV antibody testing at month 18, per Kenya guidelines. Mothers without HIV underwent HIV re‐testing at 6, 12 and 24 months postpartum. Any mother or infant newly diagnosed with HIV was referred to the HIV Care Clinic. Women or children who were diagnosed with HIV infection during 2‐year follow‐up continued to participate in the study.

### 2.5 Statistical Analysis

We examined associations between maternal characteristics and child growth outcomes. *Z*‐scores were calculated using the WHO Child Growth Standards Anthro package (version 1.0.0) in R. Length measurements at follow‐up visits that were ≤ 0.5 cm shorter than the previous visit were considered implausible and excluded. Additionally, z‐scores above or below the predefined WHO ‘flags’ (−6 < LAZ > 6, −6 < WAZ > 5, −5 < WLZ > 5) were compared to other values for the same child and excluded if deemed implausible.

A mixed effects spline model was used to examine trajectories of continuous outcomes (WAZ, LAZ and WLZ) from birth to 2 years in three stages. First, locally weighted scatterplot smoothing (LOESS) curves were fitted to visualise the outcome trajectories over time. Second, guided by the LOESS curves, piecewise linear mixed‐effect models with knots at 3 and 6 months were compared to account for observed nonlinear growth patterns. A fixed knot at Month 3 was selected as the best knot placement for modelling the outcome trajectories based on the Akaike information criterion (AIC) values for primary outcome (WAZ) and its alignment with the LOESS trajectory. Finally, the 3‐month knot was used to fit the final piecewise spline models for each exposure. with interaction terms between time (child age in months), the determinants of interest, and linear splines for time (child age in months) with a knot at month 3. For each determinant, we estimated the difference in monthly WAZ, LAZ and WLZ before and after month 3 and used the *p*‐value of the interaction term(s) to determine the statistical significance of the association between each determinant and the monthly rate of change in WAZ/LAZ/WLZ in early versus later infancy (< 3 vs. > 3 months). The models were adjusted for the following variables selected a priori: maternal age (years), wealth index (a grouped linear variable: 4 = lowest quintile, 3 = second lowest quintile, 2 = third [middle] quintile, 1 = fourth quintile, 0 = fifth [highest] quintile), gestational age (in weeks), infant sex, birth weight (for WAZ and WLZ) and birth length (for LAZ) as these factors have been associated with infant growth in previous studies (Li et al. 2020a; Mertens et al. 2023). Notably, we excluded continuous maternal age from models analysing young maternal age as a determinant and gestational age from models for preterm birth.

Unadjusted and adjusted Poisson regression models with robust variance estimation (Zou 2004) were used to assess associations between maternal determinants and binary outcomes (underweight, stunting, wasting) before 3 months and after 3 months as this was the time point identified previously. All statistical tests were two‐sided with a significance level of 5%. No adjustments for multiple comparisons were made given the exploratory nature of this study.

### 2.6 Ethics Statement

The study was approved by the Kenya Medical Research Institute Scientific and Ethics Review Unit (0140/3940) and the University of Washington Institutional Review Board (STUDY00007708).

## 3 Results

The Tunza Mwana cohort study enrolled 350 pregnant women between November 2021 and February 2022, including 175 living without HIV and 175 WLWH. Of these, 326 children attended the 24‐month follow‐up visit and had data available for the primary outcome, WAZ, and were therefore included in the analysis (Tables 1, 2; Supporting Information: Figure S1).

**Table 1:** Characteristics of pregnant women at enrolment in the Tunza Mwana birth cohort study, Kenya.

Maternal household and clinical characteristics^a^	*N* = 326
Study site	
Migori county	183 (56.1%)
St. Joseph Mission Hospital	143 (43.9%)
Age (years)	26.9 (22.8, 32.6)
Completed primary education or below	210 (64.4%)
Married	282 (84.7%)
Food insecurity	277 (85.0%)
Secure	97 (29.8%)
Mild	37 (11.3%)
Moderate	106 (32.5%)
Severe	83 (25.5%)
Minimum dietary diversity score	4 (4, 5)
Wealth index	
Quintile 1	68 (20.8%)
Quintile 2	67 (20.6%)
Quintile 3	63 (19.3%)
Quintile 4	66 (20.2%)
Quintile 5	62 (19.1%)
Improved household sanitation^b^	244 (74.8%)
Improved source of household drinking water^c^	263 (80.7%)
Household crowding (> 3 people/room)	35 (10.7%)
Receiving financial support from the child's father	289 (88.7%)
Depressive symptoms, PHQ‐9 Score > 5	2 (0, 5)
Intimate partner violence	50 (15.3%)
Adverse childhood experiences score ≥ 2	91 (27.9%)
Mid‐upper arm circumference (MUAC) (cm)	27.0 (25.3, 29.5)
Height (cm)	162 (157, 166)
Gestational age at enrolment	32.5 (31.0, 36.0)
Anaemia in pregnancy (< 11.0 g/dL)	99 (30.4%)
Multiparous	259 (79.4%)
Hospitalised during pregnancy	18 (5.5%)
Complicated pregnancy^d^	94 (28.8%)
Taking iron/folate supplement during pregnancy	287 (88.0%)
Maternal HIV characteristics	
Women living with HIV	167 (51.2%)
ART initiation before pregnancy	127 (76.0%)

^a^ Values are *n* (%) or median (IQR).

^b^ Improved sanitation includes flush or pour‐flush to a piped sewer system, septic tank pit latrines, ventilated‐improved pit latrines or pit latrines with slab or composting toilets.

^c^ Improved water sources include household connections, public standpipes, boreholes, protected dug wells, protected springs and rainwater collection.

^d^ Defined as having hypertension, preeclampsia, eclampsia, infection or bleeding during pregnancy.

**Table 2:** Infant birth and nutritional characteristics.

Birth and infant characteristics	*N* = 326
Female	154 (47.2%)
Gestational age (weeks) at birth	39.0 (38.0, 40.0)
Preterm birth (< 37 weeks)	31 (9.5%)
Weight (kg) within 7 days of birth	3.2 (2.9, 3.6)
Low birth weight (< 2.5 kg)	18 (5.5%)
Small vulnerable newborn^a^	58 (17.8%)
Length (cm) within 7 days of birth	49.6 (48.3, 51.0)
HIV exposed uninfected	167 (51.4%)
Among HIV exposed, uninfected: ARV prophylaxis from birth	
Nevirapine plus zidovudine	131 (78.4%)
Zidovudine or nevirapine alone	7 (7.2%)
Duration of breastfeeding (months)	16 (12, 19)
Exclusive breastfeeding for 6 months	181 (55.5%)
Breastfeeding at 12 months	289 (88.7%)
Child hospitalised with diarrhoea or diagnosis of malaria or any respiratory tract infection	26 (7.9%)
Growth characteristics prior to 3 months	
Underweight	36 (11.0%)
Stunted	52 (16.0%)
Wasted	45 (13.8%)
Growth characteristics after 3 months	
Underweight	51 (15.6%)
Stunted	130 (39.9%)
Wasted	29 (8.9%)

*Note:* Values are *n* (%) or median (IQR).

^a^ Children who were born preterm, small‐for‐gestational age (SGA), or with low birth weight (LBW).

### 3.1 WAZ, LAZ and WLZ Growth Trajectories

**Figure 1:**
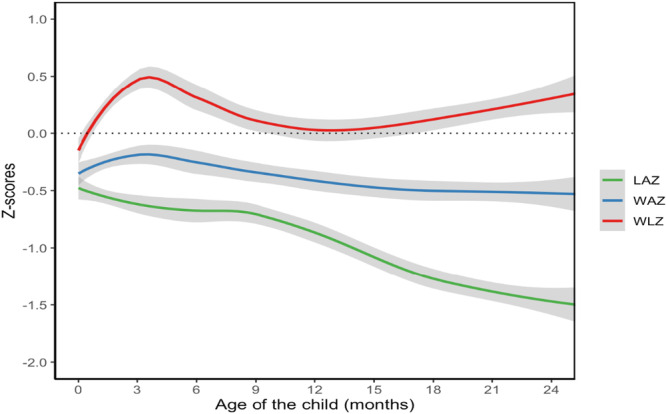
Smoothed growth trajectories of children from birth to 24 months. Locally weighted scatterplot smoothing (LOESS) curves were fitted for WAZ, LAZ and WLZ from birth to 24 months to inform piecewise linear mixed‐effect models.

**Figure 2:**
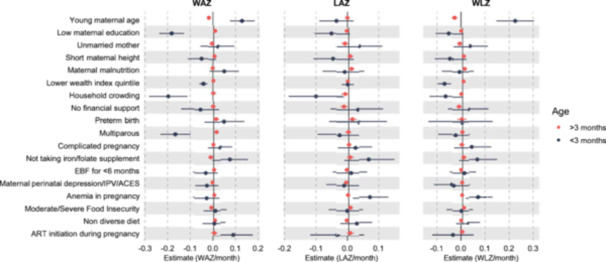
Forest plots of risk factors and child growth (WAZ, LAZ, WLZ) before and after 3 months of age. We fitted a piece‐wise linear mixed‐effects regression model with interaction terms between time (child age in months) and the determinants of interest, linear splines for time (child age in months) with a knot at Month 3 and the determinant of interest. For each determinant, we estimated the differences in *z*‐scores per month before Month 3 and after Month 3 and used the *p*‐value of the interaction term(s) to determine the statistical significance of the association between each determinant and the change in monthly WAZ, LAZ, WLZ. Models were adjusted for maternal age, wealth index, gestational age at birth, infant sex, birth weight (WAZ) and birth length (LAZ).

Figure 1 shows the LOESS curves of WAZ, LAZ and WLZ from birth to 24 months. Mean WAZ increased to a peak at 3 months before declining steadily through 24 months. LAZ declined consistently over time, while WLZ rose sharply to a peak at 3 months, dropped to a nadir at 12 months, and then began to recover (Figure 1). Results of piecewise linear mixed‐effect models with a knot at month 3, identified several maternal demographic characteristics that were associated with infant growth trajectories Younger maternal age was associated with increased monthly rate of change in WAZ before 3 months (0.13; 95% CI: 0.08 to 0.18) but decreased monthly rate of change in WAZ after 3 months (−0.02; 95% CI: −0.03 to −0.01) (Figure 2, Supporting Information: Table S2). Multiparity was associated with decreased monthly rate of change in WAZ before 3 months (−0.16; 95% CI: −0.23 to −0.1) and increased monthly rate of change in WAZ after 3 months (0.01; 95% CI: 0.00 to 0.02). Household crowding (−0.19; 95% CI: −0.28 to −0.11) and lower wealth quintile (−0.04; 95% CI: −0.06 to −0.02) were associated with decreased monthly rate of change in WAZ before 3 months. After 3 months, moderate/severe food insecurity (−0.01, 95% CI: −0.02 to 0.00) and absence of iron‐folate supplementation (−0.01; 95% CI: −0.02 to −0.001) were associated with decreased monthly rate of change in WAZ.

Household crowding was associated with decreased monthly rate of change in LAZ before 3 months (−0.10; 95% CI: −0.19 to 0.01) (Figure 2, Supporting Information: Table S2). After 3 months, being unmarried (−0.01; 95% CI: −0.023 to −0.001) and lacking financial support from a partner (−0.02; 95% CI: −0.03 to −0.00) were associated with decreased monthly rate of change in LAZ. In contrast, maternal anaemia in pregnancy was associated with increased monthly rate of change in LAZ before 3 months (0.07; 95% CI: 0.01 to 0.13).

Younger maternal age was associated with increased monthly rate of change in WLZ before 3 months (0.23; 95% CI: 0.15 to 0.30) but decreased monthly rate of change in WLZ after 3 months (−0.03; 95% CI: −0.04 to −0.01) (Figure 2, Supporting Information: Table S3). Lower wealth quintile was associated with decreased monthly rate of change in WLZ before 3 months (−0.07; 95% CI: −0.09 to −0.04). After 3 months, being unmarried (−0.01; 95% CI: −0.02 to −0.00) and having no financial support from their partner (−0.01; 95% CI: −0.03 to −0.00) were associated with decreased monthly rate of change in WLZ. Maternal anaemia in pregnancy was associated with increased monthly rate of change in WLZ (0.07; 95% CI: 0.01 to 0.13) before 3 months.

Among WLWH, ART initiation during pregnancy was associated with increased in WAZ (0.09; 95% CI: 0.003 to 0.18) in early infancy (< 3 months) compared to children whose mothers initiated ART before pregnancy (Figure 2).

### 3.2 Underweight, Stunting and Wasting

During the first 3 months of life, 11.0% children were underweight, 16.0% were stunted and 13.8% were wasted. Between 3 and 24 months, 15.6% were underweight, 39.9% were stunted and 8.9% were wasted (Table 2).

Among all the maternal factors investigated, lower gestational age and preterm birth were associated with increased risk of underweight before 3 months in adjusted and unadjusted models and after 3 months in adjusted models (Table 3). Lower wealth quintile and lack of financial support from the father were also associated with increased risk of underweight after 3 months in unadjusted models, but this was attenuated in adjusted models (Table 3). Increased maternal MUAC was associated with decreased risk of underweight after 3 months only in unadjusted models.

**Table 3:** Associations of characteristics of mother and children enrolled in the Tunza Mwana study, Kenya, with underweight.

Characteristics	Early underweight (≤ 3 months)				Underweight at > 3 months			
	RR (95% CI)	*p‐* value	aRR (95% CI)	*p‐* value	RR (95% CI)	*p‐* value	aRR (95% CI)	*p‐* value
Sociodemographic information								
Age < 25 years	0.93 (0.49, 1.77)	0.832	0.90 (0.47, 1.74)	0.761	0.62 (0.35, 1.11)	0.107	0.9 (0.47, 1.74)	0.761
Primary education or below	1.1 (0.57, 2.13)	0.766	0.98 (0.48, 2.02)	0.958	1.21 (0.70, 2.09)	0.497	0.98 (0.48, 2.02)	0.958
Unmarried^a^	1.13 (0.5, 2.57)	0.770	1.20 (0.52, 2.76)	0.673	1.05 (0.53, 2.10)	0.886	1.2 (0.52, 2.76)	0.673
Maternal anthropometry								
Shorter height^b^	1.51 (0.79, 2.88)	0.209	1.89 (0.95, 3.76)	0.069	1.26 (0.73, 2.18)	0.408	1.89 (0.95, 3.76)	0.069
MUAC, cm	0.97 (0.88, 1.08)	0.603	0.96 (0.86, 1.07)	0.441	0.88 (0.80, 0.97)	**0.008**	0.96 (0.86, 1.07)	0.441
Household factors								
Lower wealth quintile	1.02 (0.83, 1.26)	0.838	0.97 (0.8, 1.19)	0.802	1.22 (1.02, 1.47)	**0.031**	0.97 (0.8, 1.19)	0.802
Household crowding^c^	1.34 (0.56, 3.22)	0.512	1.24 (0.49, 3.11)	0.647	1.55 (0.79, 3.02)	0.201	1.24 (0.49, 3.11)	0.647
No financial support the father	1.26 (0.52, 3.04)	0.607	1.23 (0.52, 2.86)	0.637	1.91 (1.04, 3.47)	**0.035**	1.23 (0.52, 2.86)	0.637
Obstetric history and medical history								
Gestational age (weeks)	0.84 (0.73, 0.97)	**0.014**	0.85 (0.74, 0.97)	**0.015**	0.99 (0.87, 1.13)	0.920	0.85 (0.74, 0.97)	**0.015**
Preterm birth^d^	2.72 (1.36, 5.44)	**0.005**	2.77 (1.39, 5.50)	**0.004**	1.51 (0.75, 3.07)	0.250	2.77 (1.39, 5.5)	**0.004**
Multiparous	0.75 (0.37, 1.52)	0.429	0.46 (0.20, 1.08)	0.076	0.82 (0.45, 1.47)	0.496	0.46 (0.2, 1.08)	0.076
Complicated pregnancy^e^	0.73 (0.36, 1.50)	0.394	0.74 (0.37, 1.50)	0.408	0.68 (0.37, 1.24)	0.203	0.74 (0.37, 1.5)	0.408
No iron/folate supplementation	0.92 (0.34, 2.46)	0.868	0.78 (0.3, 2.05)	0.613	0.98 (0.45, 2.15)	0.962	0.78 (0.3, 2.05)	0.613
Breastfeeding								
Exclusive breastfeeding < 6 mo	1.19 (0.62, 2.27)	0.608	1.24 (0.66, 2.32)	0.505	1.38 (0.81, 2.35)	0.231	1.24 (0.66, 2.32)	0.505
Psychosocial factors								
Perinatal depression/anxiety/IPV/ > 2 ACEs^f^	0.96 (0.52, 1.79)	0.908	0.92 (0.51, 1.65)	0.772	0.9 (0.54, 1.49)	0.674	0.92 (0.51, 1.65)	0.772
Maternal nutrition and food insecurity								
Anaemia (< 11 g/dL)	1.00 (0.51, 1.96)	0.989	0.98 (0.5, 1.94)	0.961	1.21 (0.71, 2.05)	0.483	0.98 (0.5, 1.94)	0.961
Moderate/Severe Food Insecurity	1.13 (0.61, 2.10)	0.702	1.15 (0.63, 2.10)	0.658	1.47 (0.89, 2.42)	0.135	1.15 (0.63, 2.1)	0.658
Minimum dietary diversity score	0.98 (0.78, 1.22)	0.839	0.97 (0.78, 1.22)	0.819	0.93 (0.79, 1.11)	0.428	0.97 (0.78, 1.22)	0.819
Maternal HIV characteristics								
ART initiation during pregnancy	0.75 (0.27, 2.09)	0.579	0.86 (0.32, 2.30)	0.759	0.86 (0.4, 1.82)	0.684	0.86 (0.32, 2.3)	0.759

*Note: Reference categories:* ≥ 25 years (age); some secondary education or higher (education); married (marital status); ≥ 157.3 cm (height); higher wealth quintile; no crowding (≤ 3 persons/room); receiving financial support from father; ≥ 37 weeks' gestation (preterm birth); no prior birth (parity); ≥ 6 months exclusive breastfeeding; food secure/mild food insecurity; ART initiation before conception. RR or adjusted RR were obtained using Poisson regression with robust variance. Adjusted models included a priori covariates: maternal age (years), wealth index (0–4, highest to lowest quintile), gestational age (weeks) and infant sex. Models for age excluded continuous age; models for wealth excluded the wealth index; and models for gestational age and preterm birth excluded continuous gestational age. Bold values indicate statistically significant.

Abbreviations: ACEs, adverse childhood experience; ART, antiretroviral therapy; GAD‐7, generalised anxiety disorder 7‐item; MUAC, mid‐upper arm circumference; PHQ‐9, patient health questionnaire‐9; RR, relative risk.

^a^ Unmarried includes divorced/separated/living together.

^b^ Shorter height defined as < 1st quartile 157.3 cm.

^c^ Household crowding defined as > 3 people/room in house.

^d^ Preterm birth defined as < 37 weeks of gestation.

^e^ Complicated pregnancy includes infection, bleeding, high blood pressure, preeclampsia or COVID during pregnancy, or hospitalised during pregnancy).

^f^ Perinatal depression defined as PHQ9 score ≥ 5; anxiety GAD7 score ≥ 5.

Maternal height < 157.3 cm was associated with elevated risk of stunting before (aRR 2.33; 95% CI: 1.36 to 1.22) and after 3 months of life (aRR 1.36; 95% CI: 1.04 to 1.79) in unadjusted and adjusted models (Supporting Information: Table S5). Furthermore, being in a lower wealth quintile was associated with an increased risk of stunting after 3 months (aRR 1.12; 95% CI: 1.02 to 1.23) in all models. Gestational age (aRR 0.78; 95% CI: 0.72 to 0.86) and preterm birth (aRR 2.54; 95% CI: 1.43 to 4.51) were associated with the risk of early stunting but not stunting after 3 months.

We identified a few factors significantly associated with wasting in our cohort. Only lower maternal education was associated with a decreased risk of early wasting in unadjusted and adjusted models (aRR 0.5; 95% CI: 0.27 to 0.93) (Supporting Information: Table S6). None of the other factors investigated were significantly associated with child wasting.

## 4 Discussion

Several maternal characteristics were associated with child growth in this exploratory analysis of mother‐infant pairs in the Tunza Mwana cohort.

We identified differences in maternal factors associated with early (< 3 months) and later growth (> 3 months) among children in this cohort. This is not entirely surprising as early growth is influenced by events and exposures that occur in utero (Kitsiou‐Tzeli and Tzetis 2017; Strauss 1997). Young maternal age was associated with improved growth in early infancy (< 3 months) and worse growth in later infancy (> 3 months), which may reflect increased support for adolescent and young mothers in the early postpartum period and waning of this support over time. Catch‐up growth may also contribute to these differences as young maternal age is associated with increased risk of preterm birth (Gebreegziabher et al. 2023; Kozuki et al. 2013) and SGA infants (Kozuki et al. 2013; Suarez‐Idueta et al. 2021), and these infants often experience catch‐up growth patterns (Su et al. 2025). Previous studies have reported that pregnant adolescents and young women are at high risk of undernutrition (Nguyen et al. 2017), have decreased responsiveness to nutritional supplementation, potentially due to nutrient partitioning (Koroma et al. 2023) and have an increased risk of having underweight and smaller infants (Nguyen et al. 2017; Welch et al. 2024). Programs and policies targeting pregnant and lactating adolescents and young women in the first 2 years postpartum could help improve the growth outcomes of their infants. Additional studies focusing on optimal intervention strategies for this high‐risk group are needed.

Anaemia in pregnancy was associated with increased linear and weight‐for‐length growth in early infancy among children in our cohort. Prior studies have reported conflicting findings, with some observing an association between maternal anaemia and an increased risk of stunting (Nadhiroh et al. 2023), while others found no association (Heesemann et al. 2021). These conflicting findings may be attributable to heterogeneity in study designs, variations in the timing of maternal anaemia assessment, differences in the underlying prevalence of maternal anaemia across study populations, or chance findings. Furthermore, our study performed an assessment of anaemia in the third trimester at the time of enrolment, and we are unable to identify the etiology of anaemia. Anaemia and iron deficiency prevalence increases in the third trimester when most fetal iron accretion occurs, even in settings with routine iron supplementation and adequate diets (McCarthy et al. 2024). Pregnancy anaemia is associated with an increased risk of SGA and preterm infants (Chen et al. 2024; Col Madendag et al. 2019), thus our finding of an association between maternal anaemia and higher LAZ and WLZ gain prior to 3 months of life may reflect catch‐up growth among smaller infants. Iron and other micronutrients are critical for fetal and early infant growth, and a recent meta‐analyses have demonstrated multiple micronutrient supplements to provide good protection from anaemia in pregnancy (Gomes et al. 2022) and improved infant anthropometrics from birth to 6 months compared to iron‐folic acid supplements (Gomes et al. 2025). Bolstering multiple micronutrient supplement interventions among at‐risk populations should continue to be a priority for programming and policy.

We found significant associations with household assets and early infant growth. The lower wealth index quintile was associated with lower monthly WAZ and WLZ growth in early infancy. Of all the determinants investigated, only household crowding was associated with lower monthly WAZ and LAZ gains and borderline lower WLZ gain (−0.01; 95% CI: −0.01 to 0), and this was only prior to 3 months of age. Household crowding has previously been described as associated with higher total and activity energy expenditure among infants in Brazil, which may result from decreased sleep or increased infections (Haisma et al. 2006). It is important to note that the effect size of these associations was relatively small; however, as this factor was associated with all 3 growth measures, additional studies elucidating mechanisms and effective interventions are warranted. Maternal education was associated with WAZ in our study, even after adjustment for household wealth. This has been previously described in Kenya (Abuya et al. 2011) and other low‐ and middle‐income country settings (Le and Nguyen 2020; Rezaeizadeh et al. 2024). It may be driven by factors such as improved family planning, increased household wealth and resources (though this was adjusted for in our analysis), greater recognition of optimal nutritional practices, early identification of illnesses that could contribute to growth faltering, and increased maternal autonomy (Casale et al. 2018; Frost et al. 2005). Cross‐sectoral interventions aimed at strengthening household resources and promoting maternal education may represent additional pathways to support improvements in child growth.

We did not identify any significant associations between maternal mental health in pregnancy, assessed by PHQ‐9 and GAD‐7, intimate partner violence or adverse childhood experiences with infant growth outcomes in our cohort. Prior studies had reported associations between maternal mental disorders and early child growth (Bennett et al. 2016), though a meta‐analyses of interventions targeting perinatal maternal mental health have demonstrated very modest effects on child growth (Tol et al. 2020). Our dataset only included PHQ‐9 and GAD‐7 assessment in pregnancy, potentially limiting the identification of associations. Only a few studies have recently investigated associations of maternal adverse childhood experiences with child growth, as they may result in epigenetic changes, but have not found clear associations (Chung et al. 2023). Additionally, we saw a suggestion that perinatal mental health, intimate partner violence and adverse childhood experiences are associated with lower WAZ, LAZ and WLZ change after 3 months though all of these confidence intervals crossed the null. This borderline finding may be the result of insufficient sample size to detect this difference, as a few large analyses have demonstrated associations between intimate partner violence and stunting (Chai et al. 2016; Neamah et al. 2018; Ziaei et al. 2014). As studies examining the influence of maternal mental health, adverse childhood experiences and intimate partner violence on child growth remain limited, there is need for larger‐scale cohort studies or meta‐analyses of longitudinal cohorts that incorporate comprehensive assessments of these maternal factors at multiple time points to more clearly elucidate its impact on child growth outcomes. As expected, lower gestational age at birth and preterm birth were associated with increased risk of early and later underweight and early stunting after adjustment for birth anthropometrics, consistent with prior studies (Christian et al. 2013; Sania et al. 2015; Sartika et al. 2021). Maternal anthropometric characteristics were associated with the risk of stunting and underweight. Larger maternal MUAC in the third trimester was associated with a decreased risk of stunting after 3 months and underweight in unadjusted models after 3 months. Maternal prepregnancy BMI and MUAC has been identified as an important determinant of WAZ, LAZ and WLZ trajectories in similar settings (Bengtson et al. 2022; Deierlein et al. 2011; Haque et al. 2021; Kpewou et al. 2020; Zalbahar et al. 2016). Furthermore, shorter maternal height was associated with increased risk of stunting at both times, which has been well established risk factor in large multi‐country studies (Li et al. 2020b; Wu et al. 2021). Preconception and early pregnancy nutritional supplementation have been shown to improve infant ponderal and linear growth, highlighting the importance of maternal nutrition before and during early gestation and the need for interventions targeting maternal nutrition in fragile contexts (Krebs et al. 2021; Von Salmuth et al. 2021). The pathways resulting in these outcomes may involve direct macro‐ and micronutrient provision, alterations of the maternal and neonatal microbiota (García‐Mantrana et al. 2020), and epigenetic changes (Kitsiou‐Tzeli and Tzetis 2017), all of which can influence infant growth trajectories.

We identified a few factors associated with wasting. Surprisingly, we found that lower maternal education was associated with a lower risk of wasting before 3 months. This is in contrast to several large multi‐country studies reporting increased maternal education to be associated with a decreased risk of wasting (Asebe et al. 2024; Tamir et al. 2025). Our finding of an association had a wide confidence interval and may be due to residual confounding or a small sample size, especially since no association is seen after 3 months.

Our study has several strengths. The prospective longitudinal nature of the data allows us to assess temporal relationships between the exposures and growth outcomes. Data was collected using standardised data instruments, and growth monitoring occurred regularly throughout the first 24 months using rigorous protocols and standard measurement tools by trained study staff. We had excellent follow‐up of participants in the cohort with > 90% retention at 24 months. Furthermore, our data set included a comprehensive set of maternal factors including mental health, intimate partner violence and adverse childhood experiences that have not routinely been collected in large cohorts (Mertens et al. 2023). Additionally, we investigated differences in associations in early (< 3 months) and later (> 3 months) childhood instead of a single time point. Nevertheless, there are several limitations to our study. These findings reflect outcomes from a rural population in Western Kenya that accessed antenatal care and consented to participate in a 2‐year cohort study. As such, these results may not be generalisable to populations in other geographic or socio‐cultural contexts. We had a very high rate of exclusive breastfeeding and continued breastfeeding beyond 12 months postpartum, likely influenced by the Tunza Mwana study's focus on human milk composition, which may have contributed to improved child growth outcomes in this population compared to other populations. Additionally, our sample size of 326 mother‐infant pairs and population homogeneity may not provide sufficient power to identify all relevant associations. Gestational age was determined in the cohort using the LMP, not first‐trimester ultrasound, which may have resulted in incorrect estimation of gestational age. We did not correct for multiple comparisons in our analysis given the exploratory nature, so some of the significant findings may have emerged by chance; therefore, our findings must be interpreted with caution and confirmed in larger cohorts. Additionally, it is important to note that the effect size of some of the associations was relatively small and must be considered when contemplating designing new intervention programs for child growth. Finally, we are only able to report associations and cannot directly determine causality.

In conclusion, child growth trajectories in the first 2 years of life were influenced by a complex set of factors including maternal nutritional, health, demographic and socio‐economic factors, many of which are not routinely addressed in current child growth programs. By identifying maternal factors that were consistently associated with various forms of undernutrition, and those that were specific to particular outcomes or time periods, this may inform the development of more targeted and timely prevention programs. There is need for innovative, comprehensive programs that integrate nutritional, socio‐economic and household‐level strategies to optimise growth outcomes and enable children to reach their full potential. These include routine maternal nutrition screening during pregnancy, targeted support for women with low MUAC, and interventions aimed at reducing household crowding and improving maternal education. Sustained investments in adolescent health and female education also remain essential to reducing intergenerational stunting and underweight.

## Author Contributions

David Taylor Hendrixson, Christine J. McGrath, Philip James and Tanya Khara designed the research. David Taylor Hendrixson, Ruchi Tiwari and Christine J. McGrath conducted the research. David Taylor Hendrixson, Ruchi Tiwari and Christine J. McGrath analysed data or performed statistical analysis. David Taylor Hendrixson, Ruchi Tiwari, Philip James, Zulfiqar A. Bhutta, André Briend, Sheila Isanaka, Tanya Khara, Natasha Lelijveld, Mark J. Manary, Andrew Mertens, Sophie E. Moore, Kieran S. O'Brien, Jonathan Wells, Jonathan Wells, Eric Ochola, Eric Ochola and Christine J. McGrath interpreted the results. David Taylor Hendrixson wrote the first draft of the paper. David Taylor Hendrixson and Christine J. McGrath had primary responsibility for the final content. All authors have read and approved the final manuscript.

## Conflicts of Interest

The authors declare no conflicts of interest.

## Supporting information

Supporting File 1

Supporting File 2

## Data Availability

The data that support the findings of this study are available on request from the corresponding author. The data are not publicly available due to privacy or ethical restrictions.

## References

[mcn70195-bib-0001] Abuya, B. A. , E. O. Onsomu , J. K. Kimani , and D. Moore . 2011. “Influence of Maternal Education on Child Immunization and Stunting in Kenya.” Maternal and Child Health Journal 15, no. 8: 1389–1399. 10.1007/s10995-010-0670-z. 20848172

[mcn70195-bib-0002] Amadu, I. , A.‐A. Seidu , E. Duku , et al. 2021. “Risk Factors Associated With the Coexistence of Stunting, Underweight, and Wasting in Children Under 5 From 31 Sub‐Saharan African Countries.” BMJ Open 11, no. 12: e052267. 10.1136/bmjopen-2021-052267. PMC8689177 34930735

[mcn70195-bib-0003] Asebe, H. A. , Z. A. Asmare , K. U. Mare , et al. 2024. “The Level of Wasting and Associated Factors Among Children Aged 6–59 Months in Sub‐Saharan African Countries: Multilevel Ordinal Logistic Regression Analysis.” Frontiers in Nutrition 11: 1336864. 10.3389/fnut.2024.1336864. 38903623 PMC11187342

[mcn70195-bib-0004] Bengtson, A. M. , S. M. le Roux , T. K. Phillips , et al. 2022. “Relationship Between Pre‐Pregnancy Maternal Body Mass Index and Infant Weight Trajectories in HIV‐Exposed and HIV‐Unexposed Infants.” Paediatric and Perinatal Epidemiology 36, no. 4: 536–547. 10.1111/ppe.12825. 34859468 PMC9163208

[mcn70195-bib-0005] Bennett, I. M. , W. Schott , S. Krutikova , and J. R. Behrman . 2016. “Maternal Mental Health, and Child Growth and Development, in Four Low‐Income and Middle‐Income Countries.” Journal of Epidemiology and Community Health 70, no. 2: 168–173. 10.1136/jech-2014-205311. 26359503 PMC5392254

[mcn70195-bib-0006] Casale, D. , G. Espi , and S. A. Norris . 2018. “Estimating the Pathways Through Which Maternal Education Affects Stunting: Evidence From an Urban Cohort in South Africa.” Public Health Nutrition 21, no. 10: 1810–1818. 10.1017/S1368980018000125. 29455701 PMC10260984

[mcn70195-bib-0007] Chai, J. , G. Fink , S. Kaaya , et al. 2016. “Association Between Intimate Partner Violence and Poor Child Growth: Results From 42 Demographic and Health Surveys.” Bulletin of the World Health Organization 94, no. 5: 331–339. 10.2471/BLT.15.152462. 27147763 PMC4850526

[mcn70195-bib-0008] Chen, Y. , T. Zhong , X. Song , et al. 2024. “Maternal Anaemia During Early Pregnancy and the Risk of Neonatal Outcomes: A Prospective Cohort Study in Central China.” BMJ Paediatrics Open 8, no. 1: e001931. 10.1136/bmjpo-2023-001931. 38233082 PMC10806529

[mcn70195-bib-0009] Chowdhury, M. R. K. , M. S. Rahman , B. Billah , R. Kabir , N. K. P. Perera , and M. Kader . 2022. “The Prevalence and Socio‐Demographic Risk Factors of Coexistence of Stunting, Wasting, and Underweight Among Children Under Five Years in Bangladesh: A Cross‐Sectional Study.” BMC Nutrition 8, no. 1: 84. 10.1186/s40795-022-00584-x. 35996184 PMC9394024

[mcn70195-bib-0010] Christian, P. , S. E. Lee , M. Donahue Angel , et al. 2013. “Risk of Childhood Undernutrition Related to Small‐For‐Gestational Age and Preterm Birth in Low‐ and Middle‐Income Countries.” International Journal of Epidemiology 42, no. 5: 1340–1355. 10.1093/ije/dyt109. 23920141 PMC3816349

[mcn70195-bib-0011] Chung, E. O. , E. Scherer , K. LeMasters , et al. 2023. “Maternal Adverse Childhood Experiences on Child Growth and Development in Rural Pakistan: An Observational Cohort Study.” PLOS Global Public Health 3, no. 10: e0001669. 10.1371/journal.pgph.0001669. 37878564 PMC10599588

[mcn70195-bib-0012] Coates, J. , A. Swindale , and P. Bilinsky . 2007. Household Food Insecurity Access Scale (HFIAS) for Measurement of Household Food Access: Indicator Guide (v. 3). FHI 360/FANTA.

[mcn70195-bib-0013] Col Madendag, I. , M. Eraslan Sahin , Y. Madendag , et al. 2019. “The Effect of Iron Deficiency Anemia Early in the Third Trimester on Small for Gestational Age and Birth Weight: A Retrospective Cohort Study on Iron Deficiency Anemia and Fetal Weight.” BioMed Research International 2019: 7613868. 10.1155/2019/7613868. 31886249 PMC6893279

[mcn70195-bib-0014] Deierlein, A. L. , A. M. Siega‐Riz , L. S. Adair , and A. H. Herring . 2011. “Effects of Pre‐Pregnancy Body Mass Index and Gestational Weight Gain on Infant Anthropometric Outcomes.” Journal of Pediatrics 158, no. 2: 221–226. 10.1016/j.jpeds.2010.08.008. 20863516 PMC3017634

[mcn70195-bib-0015] FAO & FHI 360 . 2016. Minimum Dietary Diversity for Women: A Guide for Measurement. FAO.

[mcn70195-bib-0016] Frost, M. B. , R. Forste , and D. W. Haas . 2005. “Maternal Education and Child Nutritional Status in Bolivia: Finding the Links.” Social Science & Medicine (1982) 60, no. 2: 395–407. 10.1016/j.socscimed.2004.05.010. 15522494

[mcn70195-bib-0017] García‐Mantrana, I. , M. Selma‐Royo , S. González , A. Parra‐Llorca , C. Martínez‐Costa , and M. C. Collado . 2020. “Distinct Maternal Microbiota Clusters Are Associated With Diet During Pregnancy: Impact on Neonatal Microbiota and Infant Growth During the First 18 Months of Life.” Gut Microbes 11, no. 4: 962–978. 10.1080/19490976.2020.1730294. 32167021 PMC7524361

[mcn70195-bib-0018] Gebreegziabher, E. , M. Bountogo , A. Sié , et al. 2023. “Influence of Maternal Age on Birth and Infant Outcomes at 6 Months: A Cohort Study With Quantitative Bias Analysis.” International Journal of Epidemiology 52, no. 2: 414–425. 10.1093/ije/dyac236. 36617176 PMC10114123

[mcn70195-bib-0019] Gomes, F. , S. Adu‐Afarwuah , R. Agustina , et al. 2025. “Effect of Prenatal Multiple Micronutrient Supplementation Compared With Iron and Folic Acid Supplementation on Size at Birth and Subsequent Growth Through 24 mo of Age: A Systematic Review and Meta‐Analysis.” American Journal of Clinical Nutrition 122, no. 1: 185–195. 10.1016/j.ajcnut.2025.04.022. 40306386 PMC12308086

[mcn70195-bib-0020] Gomes, F. , R. Agustina , R. E. Black , et al. 2022. “Multiple Micronutrient Supplements Versus Iron‐Folic Acid Supplements and Maternal Anemia Outcomes: An Iron Dose Analysis.” Annals of the New York Academy of Sciences 1512, no. 1: 114–125. 10.1111/nyas.14756. 35218047 PMC9306935

[mcn70195-bib-0021] Haisma, H. , W. A. Coward , G. H. Visser , et al. 2006. “Socio‐Economic and Environmental Factors Influence Energy Utilization in Brazilian Breast‐Fed Infants.” Journal of Nutrition 136, no. 11: 2945–2951. 10.1093/jn/136.11.2945. 17056827

[mcn70195-bib-0022] Haque, M. A. , N. Choudhury , F. D. Farzana , et al. 2021. “Determinants of Maternal Low Mid‐Upper Arm Circumference and Its Association With Child Nutritional Status Among Poor and Very Poor Households in Rural Bangladesh.” Maternal & Child Nutrition 17, no. 4: e13217. 10.1111/mcn.13217. 34018337 PMC8476420

[mcn70195-bib-0023] Heesemann, E. , C. Mähler , M. A. Subramanyam , and S. Vollmer . 2021. “Pregnancy Anaemia, Child Health and Development: A Cohort Study in Rural India.” BMJ Open 11, no. 11: e046802. 10.1136/bmjopen-2020-046802. PMC8593731 34772744

[mcn70195-bib-0024] Kerac, M. , P. T. James , M. McGrath , et al. 2025. “Malnutrition in Infants Aged under 6 Months: Prevalence and Anthropometric Assessment – Analysis of 56 Low‐ and Middle‐Income Country DHS Datasets.” BMJ Global Health 10, no. 5: e016121. 10.1136/bmjgh-2024-016121. PMC12142141 40441741

[mcn70195-bib-0025] Khara, T. , M. Myatt , K. Sadler , et al. 2023. “Anthropometric Criteria for Best‐Identifying Children at High Risk of Mortality: A Pooled Analysis of Twelve Cohorts.” Public Health Nutrition 26, no. 4: 803–819. 10.1017/S136898002300023X. 36734049 PMC10131149

[mcn70195-bib-0026] Kitsiou‐Tzeli, S. , and M. Tzetis . 2017. “Maternal Epigenetics and Fetal and Neonatal Growth.” Current Opinion in Endocrinology, Diabetes & Obesity 24, no. 1: 43–46. 10.1097/MED.0000000000000305. 27898587

[mcn70195-bib-0027] Koroma, A. S. , M. Ellie , K. Bangura , et al. 2023. “Supplementary Feeding and Infection Control in Pregnant Adolescents‐A Secondary Analysis of a Randomized Trial Among Malnourished Women in Sierra Leone.” Maternal & Child Nutrition 19, no. 1: e13456. 10.1111/mcn.13456. 36349973 PMC9749587

[mcn70195-bib-0028] Kozuki, N. , A. C. Lee , M. F. Silveira , et al. 2013. “The Associations of Parity and Maternal Age With Small‐for‐Gestational‐Age, Preterm, and Neonatal and Infant Mortality: A Meta‐Analysis.” BMC Public Health 13, no. S3: S2. 10.1186/1471-2458-13-S3-S2. PMC3847520 24564800

[mcn70195-bib-0029] Kpewou, D. E. , E. Poirot , J. Berger , et al. 2020. “Maternal Mid‐Upper Arm Circumference During Pregnancy and Linear Growth Among Cambodian Infants During the First Months of Life.” Maternal & Child Nutrition 16 Suppl 2, no. Suppl 2: e12951. 10.1111/mcn.12951. 32835455 PMC7591302

[mcn70195-bib-0030] Krebs, N. F. , K. M. Hambidge , J. L. Westcott , et al. 2021. “Growth From Birth Through Six Months for Infants of Mothers in the ‘Women First’ Preconception Maternal Nutrition Trial.” Journal of Pediatrics 229: 199–206.e4. 10.1016/j.jpeds.2020.09.032. 32956698 PMC7855785

[mcn70195-bib-0031] Kroenke, K. , R. L. Spitzer , and J. B. W. Williams . 2001. “The PHQ‐9: Validity of a Brief Depression Severity Measure.” Journal of General Internal Medicine 16, no. 9: 606–613. 10.1046/j.1525-1497.2001.016009606.x. 11556941 PMC1495268

[mcn70195-bib-0032] Le, K. , and M. Nguyen . 2020. “Shedding Light on Maternal Education and Child Health in Developing Countries.” World Development 133: 105005. 10.1016/j.worlddev.2020.105005.

[mcn70195-bib-0033] Li, Z. , R. Kim , S. Vollmer , and S. V. Subramanian . 2020a. “Factors Associated With Child Stunting, Wasting, and Underweight in 35 Low‐ and Middle‐Income Countries.” JAMA Network Open 3, no. 4: e203386. 10.1001/jamanetworkopen.2020.3386. 32320037 PMC7177203

[mcn70195-bib-0034] Li, Z. , R. Kim , S. Vollmer , and S. V. Subramanian . 2020b. “Factors Associated With Child Stunting, Wasting, and Underweight in 35 Low‐ and Middle‐Income Countries.” JAMA Network Open 3, no. 4: e203386. 10.1001/jamanetworkopen.2020.3386. 32320037 PMC7177203

[mcn70195-bib-0035] McCarthy, E. K. , D. Schneck , S. Basu , et al. 2024. “Longitudinal Evaluation of Iron Status During Pregnancy: A Prospective Cohort Study in a High‐Resource Setting.” American Journal of Clinical Nutrition 120, no. 5: 1259–1268. 10.1016/j.ajcnut.2024.08.010. 39510727

[mcn70195-bib-0036] Mertens, A. , J. Benjamin‐Chung , J. M. Colford , et al. 2023. “Causes and Consequences of Child Growth Faltering in Low‐Resource Settings.” Nature 621, no. 7979: 568–576. 10.1038/s41586-023-06501-x. 37704722 PMC10511328

[mcn70195-bib-0037] Mwangome, M. K. , and J. A. Berkley . 2014. “The Reliability of Weight‐For‐Length/Height z Scores in Children.” Maternal & Child Nutrition 10, no. 4: 474–480. 10.1111/mcn.12124. 24785183 PMC4282477

[mcn70195-bib-0038] Myatt, M. , T. Khara , S. Schoenbuchner , et al. 2018. “Children Who Are Both Wasted and Stunted Are Also Underweight and Have a High Risk of Death: A Descriptive Epidemiology of Multiple Anthropometric Deficits Using Data From 51 Countries.” Archives of Public Health 76, no. 1: 28. 10.1186/s13690-018-0277-1. 30026945 PMC6047117

[mcn70195-bib-0039] Nadhiroh, S. R. , F. Micheala , S. E. H. Tung , and T. C. Kustiawan . 2023. “Association Between Maternal Anemia and Stunting in Infants and Children Aged 0–60 Months: A Systematic Literature Review.” Nutrition 115: 112094. 10.1016/j.nut.2023.112094. 37572547

[mcn70195-bib-0040] Neamah, H. H. , C. Sudfeld , D. C. McCoy , et al. 2018. “Intimate Partner Violence, Depression, and Child Growth and Development.” Pediatrics 142, no. 1: e20173457. 10.1542/peds.2017-3457. 29891566

[mcn70195-bib-0041] Nguyen, P. H. , T. Sanghvi , L. M. Tran , et al. 2017. “The Nutrition and Health Risks Faced by Pregnant Adolescents: Insights From a Cross‐Sectional Study in Bangladesh.” PLoS One 12, no. 6: e0178878. 10.1371/journal.pone.0178878. 28594949 PMC5464569

[mcn70195-bib-0042] Rezaeizadeh, G. , M. A. Mansournia , A. Keshtkar , et al. 2024. “Maternal Education and Its Influence on Child Growth and Nutritional Status During the First Two Years of Life: A Systematic Review and Meta‐Analysis.” EClinicalMedicine 71: 102574. 10.1016/j.eclinm.2024.102574. 38596614 PMC11001623

[mcn70195-bib-0043] Sadler, K. , P. T. James , Z. A. Bhutta , et al. 2022. “How Can Nutrition Research Better Reflect the Relationship Between Wasting and Stunting in Children? Learnings From the Wasting and Stunting Project.” Journal of Nutrition 152, no. 12: 2645–2651. 10.1093/jn/nxac091. PMC9839990 35687496

[mcn70195-bib-0044] Sadler, K. , T. Khara , and N. Sessions . 2021. *Best Practice in Preventing Child Wasting Within the Wider Context of Undernutrition*. https://www.ennonline.net/resource/wast/best-practice-preventing-child-wasting-within-wider-context-undernutrition.

[mcn70195-bib-0045] Von Salmuth, V. , E. Brennan , M. Kerac , M. McGrath , S. Frison , and N. Lelijveld . 2021. “Maternal‐Focused Interventions to Improve Infant Growth and Nutritional Status in Low‐Middle Income Countries: A Systematic Review of Reviews.” PLoS One 16, no. 8: e0256188. 10.1371/journal.pone.0256188. 34407128 PMC8372927

[mcn70195-bib-0046] Sania, A. , D. Spiegelman , J. Rich‐Edwards , et al. 2015. “The Contribution of Preterm Birth and Intrauterine Growth Restriction to Childhood Undernutrition in Tanzania.” Maternal & Child Nutrition 11, no. 4: 618–630. 10.1111/mcn.12123. 24720471 PMC4300290

[mcn70195-bib-0047] Sartika, A. N. , M. Khoirunnisa , E. Meiyetriani , E. Ermayani , I. L. Pramesthi , and A. J. Nur Ananda . 2021. “Prenatal and Postnatal Determinants of Stunting at Age 0–11 Months: A Cross‐Sectional Study in Indonesia.” PLoS One 16, no. 7: e0254662. 10.1371/journal.pone.0254662. 34260622 PMC8279365

[mcn70195-bib-0048] Soeken, K. L. , J. McFarlane , B. Parker , and M. C. Lominack . 1998. “The Abuse Assessment Screen: A Clinical Instrument to Measure Frequency, Severity, and Perpetrator of Abuse Against Women.” In Empowering Survivors of Abuse: Health Care for Battered Women and Their Children, 195–203. Sage Publications, Inc.

[mcn70195-bib-0049] Spitzer, R. L. , K. Kroenke , J. B. W. Williams , and B. Löwe . 2006. “A Brief Measure for Assessing Generalized Anxiety Disorder: The GAD‐7.” Archives of Internal Medicine 166, no. 10: 1092–1097. 10.1001/archinte.166.10.1092. 16717171

[mcn70195-bib-0050] Strauss, R. S. 1997. “Effects of the Intrauterine Environment on Childhood Growth.” British Medical Bulletin 53, no. 1: 81–95. 10.1093/oxfordjournals.bmb.a011608. 9158286

[mcn70195-bib-0051] Su, Y.‐Y. , C.‐J. Chen , M.‐H. Chen , et al. 2025. “Long‐Term Effects on Growth in Preterm and Small for Gestational Age Infants: A National Birth Cohort Study.” Pediatrics and Neonatology 66, no. 2: 168–175. 10.1016/j.pedneo.2024.06.007. 39107217

[mcn70195-bib-0052] Suarez‐Idueta, L. , H. Bedford , E.O. Ohuma , and M. Cortina‐Borja . 2021. “Maternal Risk Factors for Small‐For‐Gestational‐Age Newborns in Mexico: Analysis of a Nationwide Representative Cohort.” Frontiers in Public Health 9: 707078. 10.3389/fpubh.2021.707078. 35004559 PMC8732993

[mcn70195-bib-0053] Tamir, T. T. , A. F. Zegeye , B. S. Workneh , et al. 2025. “Childhood Wasting and Associated Factors in Africa: Evidence From Standard Demographic and Health Surveys From 35 Countries.” BMC Public Health 25, no. 1: 454. 10.1186/s12889-025-21673-z. 39905368 PMC11796205

[mcn70195-bib-0054] Tol, W. A. , M. C. Greene , M. E. Lasater , et al. 2020. “Impact of Maternal Mental Health Interventions on Child‐Related Outcomes in Low‐ and Middle‐Income Countries: A Systematic Review and Meta‐Analysis.” Epidemiology and Psychiatric Sciences 29: e174. 10.1017/S2045796020000864. 33070789 PMC7681164

[mcn70195-bib-0055] Tufts University . 2023. *Data4Diets: Building Blocks for Diet‐Related Food Security Analysis, Version 2.0*. https://inddex.nutrition.tufts.edu/data4diets.

[mcn70195-bib-0056] United Nations Children's Fund, World Health Organization, International Bank for Reconstruction and Development/The World Bank . 2025. Levels and Trends in Child Malnutrition: Key Findings of the 2025 Edition of the Joint Child Malnutrition Estimates. World Health Organization.

[mcn70195-bib-0057] Welch, C. , C. K. Wong , N. Lelijveld , M. Kerac , and S. V. Wrottesley . 2024. “Adolescent Pregnancy Is Associated With Child Undernutrition: Systematic Review and Meta‐Analysis.” Maternal & Child Nutrition 20, no. 1: e13569. 10.1111/mcn.13569. 37781871 PMC10749999

[mcn70195-bib-0058] Wells, J. , A. Briend , E. M. Boyd , et al. 2019. “Beyond Wasted and Stunted‐A Major Shift to Fight Child Undernutrition.” Lancet. Child & Adolescent Health 3, no. 11: 831–834. 10.1016/S2352-4642(19)30244-5. 31521500

[mcn70195-bib-0059] World Health Organization . 2018. Adverse Childhood Experiences International Questionnaire (ACE‐IQ). WHO.

[mcn70195-bib-0060] Wu, H. , C. Ma , L. Yang , and B. Xi . 2021. “Association of Parental Height With Offspring Stunting in 14 Low‐ and Middle‐Income Countries.” Frontiers in Nutrition 8: 650976. 10.3389/fnut.2021.650976. 34458296 PMC8384954

[mcn70195-bib-0061] Zalbahar, N. , H. J. Jan Mohamed , S. L. Loy , J. Najman , H. D. McIntyre , and A. Mamun . 2016. “Association of Parental Body Mass Index Before Pregnancy on Infant Growth and Body Composition: Evidence From a Pregnancy Cohort Study in Malaysia.” Obesity Research & Clinical Practice 10 Suppl 1: 35. 10.1016/j.orcp.2015.08.002. 26321098

[mcn70195-bib-0062] Ziaei, S. , R. T. Naved , and E. C. Ekström . 2014. “Women's Exposure to Intimate Partner Violence and Child Malnutrition: Findings From Demographic and Health Surveys in Bangladesh.” Maternal & Child Nutrition 10, no. 3: 347–359. 10.1111/j.1740-8709.2012.00432.x. 22906219 PMC6860329

[mcn70195-bib-0063] Zou, G. 2004. “A Modified Poisson Regression Approach to Prospective Studies With Binary Data.” American Journal of Epidemiology 159, no. 7: 702–706. 10.1093/aje/kwh090. 15033648

