# Supplementary material for: Maternal Health and Sociodemographic Characteristics Influence Infant Growth to 24 Months in the Tunza Mwana Cohort: A Prospective Cohort Study
Source: Matern Child Nutr. 2026 May 14;22(3):e70195. doi: 10.1111/mcn.70195 (PMC13176631; doi:10.1111/mcn.70195)
Supplement: Supplementary file 1 — Supporting File 1 [file MCN-22-e70195-s002.docx]

**Supplemental Materials**

**843 excluded**

563 were <28 or >42 weeks’ gestation

250 not willing to participate^**^

133 outside catchment area^*^

91 aged <18 and >40 years

23 previously enrolled

12 preeclampsia/hypertension

11 not planning to breastfeed

7 unknown HIV status

3 other

1442 pregnant women screened for eligibility

350 pregnant women enrolled

**24 excluded (no 24 mo growth measures)**

9 voluntary withdrawal

1 fetal death

1 congenital anomaly

3 child deaths

1 moved out of area

9 untraceable

326 included in growth analysis

**S1 Fig. Flowchart of children in the Tunza Mwana cohort included in the growth analysis.**

*Outside catchment area includes residing outside catchment area and not remaining in study area for 2 years

^**^Not willing to participate includes not willing to return for follow up, not willing to be contacted for follow up visits or have home follow up visits, not interested in participating, and not willing to provide informed consent

**Supplementary Table 1. Exposure Variable Definitions**

| **Variable Name** | **Description** |
| --- | --- |
| Age <25 years | Maternal age less than 25 years |
| Primary education or below | No or primary education and below. Reference some secondary education and higher |
| Unmarried | includes divorced/separated/living together |
| Shorter height | <1st quartile 157.3 cm |
| MUAC, cm | Mid-upper arm circumference in centimeters |
| Lower wealth quintile | Lowest wealth quintile from a group linear wealth index variable with 5 quintiles |
| Household crowding | defined as >3 people/room in house |
| No financial support the father | Does not receive financial support from the father of the child |
| Gestational age | Gestational age at birth in weeks |
| Preterm birth | <37 weeks of gestation at birth |
| Multiparous | Second or higher pregnancy |
| Complicated pregnancy | Infection, bleeding, high blood pressure, preeclampsia, or COVID during pregnancy, or hospitalized during pregnancy |
| No iron/folate supplementation | Not taking iron and folic acid supplementation in pregnancy |
| Exclusive breastfeeding <6 mo | Reported exclusive breastfeeding for less than 6 months |
| Perinatal depression/anxiety/IPV/ >2 ACEs | PHQ9 score ≥ 5; anxiety GAD7 score ≥ 5; Intimate partner violence, and >2 ACEs |
| Anemia | Third trimester hemoglobin level < 11 g/dL |
| Moderate/Severe Food Insecurity | Moderate or severe food insecurity on Household Food Insecurity Access Scale |
| Minimum dietary diversity score/ Non-diverse diet | Dietary diversity score from MDD-W. Non-diverse diet when <5 of 10 categories. |
| ART initiation during pregnancy | Began ART during pregnancy/ not on ART prior to pregnancy |

**Supplementary Table 2. Associations between maternal determinants and the monthly rate of change in weight-for-age z-score before and after 3 months of age.**

| **Time** | **Variable** | **Estimate** | **95% CI** | **p-Value** | **Standard Error** |
| --- | --- | --- | --- | --- | --- |
| <3 months | Young maternal age | 0.13 | 0.076, 0.184 | <0.001 | 0.028 |
| <3 months | Low maternal education | -0.182 | -0.236, -0.128 | <0.001 | 0.028 |
| <3 months | Unmarried mother | 0.02 | -0.054, 0.093 | 0.597 | 0.038 |
| <3 months | Short maternal height | -0.05 | -0.111, 0.011 | 0.108 | 0.031 |
| <3 months | Maternal malnutrition | 0.05 | -0.015, 0.115 | 0.128 | 0.033 |
| <3 months | Lower wealth index quintile | -0.041 | -0.06, -0.023 | <0.001 | 0.009 |
| <3 months | Household crowding | -0.197 | -0.282, -0.112 | <0.001 | 0.043 |
| <3 months | No financial support | -0.058 | -0.141, 0.024 | 0.166 | 0.042 |
| <3 months | Preterm birth | 0.049 | -0.041, 0.14 | 0.287 | 0.046 |
| <3 months | Multiparous | -0.165 | -0.231, -0.099 | <0.001 | 0.034 |
| <3 months | Complicated pregnancy | 0.03 | -0.021, 0.093 | 0.21 | 0.028 |
| <3 months | Not taking iron/folate supplement | 0.075 | -0.006, 0.155 | 0.068 | 0.041 |
| <3 months | EBF for <6 months | -0.035 | -0.087, 0.017 | 0.192 | 0.027 |
| <3 months | Maternal perinatal depression/IPV/ACES | -0.03 | -0.083, 0.023 | 0.265 | 0.026 |
| <3 months | Anemia in pregnancy | -0.031 | -0.089, 0.026 | 0.281 | 0.029 |
| <3 months | Moderate/Severe Food Insecurity | 0.008 | -0.046, 0.062 | 0.778 | 0.027 |
| <3 months | Non diverse diet | 0.003 | -0.05, 0.056 | 0.917 | 0.027 |
| <3 months | ART initiation during pregnancy | 0.09 | 0.003, 0.176 | 0.042 | 0.044 |
| >3 months | Young maternal age | -0.018 | -0.027, -0.01 | <0.001 | 0.004 |
| >3 months | Low maternal education | 0.007 | -0.001, 0.015 | 0.096 | 0.004 |
| >3 months | Unmarried mother | -0.007 | -0.018, 0.003 | 0.181 | 0.006 |
| >3 months | Short maternal height | 0.004 | -0.005, 0.013 | 0.413 | 0.005 |
| >3 months | Maternal malnutrition | -0.001 | -0.011, 0.008 | 0.795 | 0.005 |
| >3 months | Lower wealth index quintile | 0.002 | -0.001, 0.005 | 0.153 | 0.001 |
| >3 months | Household crowding | 0.002 | -0.011, 0.014 | 0.796 | 0.006 |
| >3 months | No financial support | -0.002 | -0.014, 0.01 | 0.698 | 0.006 |
| >3 months | Preterm birth | 0.011 | -0.002, 0.024 | 0.102 | 0.007 |
| >3 months | Multiparous | 0.013 | 0.003, 0.023 | 0.01 | 0.005 |
| >3 months | Complicated pregnancy | -0.001 | -0.009, 0.007 | 0.81 | 0.004 |
| >3 months | Not taking iron/folate supplement | -0.013 | -0.025, -0.001 | 0.032 | 0.006 |
| >3 months | EBF for <6 months | 0.002 | -0.006, 0.009 | 0.66 | 0.004 |
| >3 months | Maternal perinatal depression/IPV/ACES | -0.005 | -0.013, 0.002 | 0.174 | 0.003 |
| >3 months | Anemia in pregnancy | 0.003 | -0.006, 0.011 | 0.524 | 0.004 |
| >3 months | Moderate/Severe Food Insecurity | -0.01 | -0.018, -0.002 | 0.015 | 0.004 |
| >3 months | Non diverse diet | 0.006 | -0.002, 0.014 | 0.117 | 0.004 |
| >3 months | ART initiation during pregnancy | 0.003 | -0.01, 0.016 | 0.659 | 0.007 |

**Supplementary Table 3. Associations between maternal determinants and the monthly rate of change in length-for-age z-score before and after 3 months of age.**

| **Time** | **Variable** | **Estimate** | **95% CI** | **p-Value** | **Standard Error** |
| --- | --- | --- | --- | --- | --- |
| <3 months | Young maternal age | -0.035 | -0.091, 0.02 | 0.211 | 0.028 |
| <3 months | Low maternal education | -0.051 | -0.106, 0.005 | 0.074 | 0.028 |
| <3 months | Unmarried mother | 0.037 | -0.038, 0.112 | 0.328 | 0.038 |
| <3 months | Short maternal height | -0.046 | -0.108, 0.017 | 0.151 | 0.032 |
| <3 months | Maternal malnutrition | -0.013 | -0.079, 0.052 | 0.689 | 0.034 |
| <3 months | Lower wealth index quintile | -0.003 | -0.021, 0.016 | 0.791 | 0.01 |
| <3 months | Household crowding | -0.101 | -0.187, -0.014 | 0.022 | 0.044 |
| <3 months | No financial support | 0.03 | -0.054, 0.113 | 0.484 | 0.043 |
| <3 months | Preterm birth | 0.034 | -0.058, 0.126 | 0.469 | 0.047 |
| <3 months | Multiparous | -0.029 | -0.097, 0.038 | 0.398 | 0.034 |
| <3 months | Complicated pregnancy | 0.022 | -0.036, 0.08 | 0.453 | 0.029 |
| <3 months | Not taking iron/folate supplement | 0.068 | -0.014, 0.15 | 0.104 | 0.042 |
| <3 months | EBF for <6 months | 0.008 | -0.046, 0.062 | 0.775 | 0.028 |
| <3 months | Maternal perinatal depression/IPV/ACES | -0.015 | -0.068, 0.039 | 0.595 | 0.027 |
| <3 months | Anemia in pregnancy | 0.072 | 0.013, 0.131 | 0.016 | 0.03 |
| <3 months | Moderate/Severe Food Insecurity | -0.005 | -0.06, 0.049 | 0.849 | 0.028 |
| <3 months | Non diverse diet | 0.026 | -0.027, 0.08 | 0.336 | 0.027 |
| <3 months | ART initiation during pregnancy | -0.033 | -0.12, 0.053 | 0.451 | 0.044 |
| >3 months | Young maternal age | 0 | -0.008, 0.008 | 0.947 | 0.004 |
| >3 months | Low maternal education | -0.006 | -0.014, 0.003 | 0.184 | 0.004 |
| >3 months | Unmarried mother | -0.012 | -0.023, -0.001 | 0.033 | 0.006 |
| >3 months | Short maternal height | 0.005 | -0.004, 0.014 | 0.274 | 0.005 |
| >3 months | Maternal malnutrition | 0.009 | -0.001, 0.019 | 0.066 | 0.005 |
| >3 months | Lower wealth index quintile | -0.003 | -0.005, 0 | 0.06 | 0.001 |
| >3 months | Household crowding | -0.011 | -0.024, 0.001 | 0.083 | 0.006 |
| >3 months | No financial support | -0.015 | -0.027, -0.003 | 0.017 | 0.006 |
| >3 months | Preterm birth | 0.012 | -0.002, 0.025 | 0.083 | 0.007 |
| >3 months | Multiparous | -0.001 | -0.011, 0.009 | 0.78 | 0.005 |
| >3 months | Complicated pregnancy | 0.001 | -0.008, 0.01 | 0.747 | 0.004 |
| >3 months | Not taking iron/folate supplement | 0.005 | -0.007, 0.017 | 0.404 | 0.006 |
| >3 months | EBF for <6 months | -0.006 | -0.014, 0.002 | 0.136 | 0.004 |
| >3 months | Maternal perinatal depression/IPV/ACES | -0.008 | -0.015, 0.001 | 0.075 | 0.004 |
| >3 months | Anemia in pregnancy | -0.002 | -0.011, 0.006 | 0.597 | 0.004 |
| >3 months | Moderate/Severe Food Insecurity | 0.006 | -0.003, 0.014 | 0.182 | 0.004 |
| >3 months | Non diverse diet | -0.006 | -0.014, 0.002 | 0.171 | 0.004 |
| >3 months | ART initiation during pregnancy | 0.005 | -0.008, 0.018 | 0.446 | 0.006 |

**Supplementary Table 4. Associations between maternal determinants and the monthly rate of change in weight-for-length z-score before and after 3 months of age.**

| **Time** | **Variable** | **Estimate** | **95% CI** | **p-Value** | **Standard Error** |
| --- | --- | --- | --- | --- | --- |
| <3 months | Young maternal age | 0.225 | 0.148, 0.303 | <0.001 | 0.04 |
| <3 months | Low maternal education | -0.051 | -0.106, 0.005 | 0.074 | 0.028 |
| <3 months | Unmarried mother | 0.037 | -0.037, 0.112 | 0.327 | 0.038 |
| <3 months | Short maternal height | -0.046 | -0.108, 0.017 | 0.151 | 0.032 |
| <3 months | Maternal malnutrition | -0.013 | -0.079, 0.052 | 0.689 | 0.034 |
| <3 months | Lower wealth index quintile | -0.068 | -0.095, -0.042 | <0.001 | 0.014 |
| <3 months | Household crowding | -0.064 | -0.13, 0.003 | 0.061 | 0.034 |
| <3 months | No financial support | 0.03 | -0.054, 0.114 | 0.482 | 0.043 |
| <3 months | Preterm birth | -0.002 | -0.134, 0.131 | 0.982 | 0.068 |
| <3 months | Multiparous | -0.029 | -0.097, 0.038 | 0.398 | 0.034 |
| <3 months | Complicated pregnancy | 0.045 | -0.037, 0.126 | 0.284 | 0.041 |
| <3 months | Not taking iron/folate supplement | 0.068 | -0.014, 0.15 | 0.104 | 0.042 |
| <3 months | EBF for <6 months | 0.008 | -0.046, 0.062 | 0.775 | 0.028 |
| <3 months | Maternal perinatal depression/IPV/ACES | -0.037 | -0.113, 0.039 | 0.343 | 0.039 |
| <3 months | Anemia in pregnancy | 0.072 | 0.013, 0.131 | 0.016 | 0.03 |
| <3 months | Moderate/Severe Food Insecurity | -0.005 | -0.06, 0.049 | 0.846 | 0.028 |
| <3 months | Non diverse diet | 0.026 | -0.027, 0.08 | 0.336 | 0.027 |
| <3 months | ART initiation during pregnancy | -0.033 | -0.12, 0.053 | 0.451 | 0.044 |
| >3 months | Young maternal age | -0.026 | -0.037, -0.014 | 0 | 0.006 |
| >3 months | Low maternal education | -0.006 | -0.014, 0.003 | 0.184 | 0.004 |
| >3 months | Unmarried mother | -0.012 | -0.023, -0.001 | 0.033 | 0.006 |
| >3 months | Short maternal height | 0.005 | -0.004, 0.014 | 0.274 | 0.005 |
| >3 months | Maternal malnutrition | 0.009 | -0.001, 0.019 | 0.066 | 0.005 |
| >3 months | Lower wealth index quintile | 0.004 | 0, 0.008 | 0.077 | 0.002 |
| >3 months | Household crowding | -0.005 | -0.015, 0.005 | 0.312 | 0.005 |
| >3 months | No financial support | -0.015 | -0.027, -0.003 | 0.017 | 0.006 |
| >3 months | Preterm birth | -0.003 | -0.022, 0.016 | 0.761 | 0.01 |
| >3 months | Multiparous | -0.001 | -0.011, 0.009 | 0.78 | 0.005 |
| >3 months | Complicated pregnancy | -0.003 | -0.015, 0.009 | 0.617 | 0.006 |
| >3 months | Not taking iron/folate supplement | 0.005 | -0.007, 0.017 | 0.403 | 0.006 |
| >3 months | EBF for <6 months | -0.006 | -0.014, 0.002 | 0.136 | 0.004 |
| >3 months | Maternal perinatal depression/IPV/ACES | -0.002 | -0.013, 0.009 | 0.758 | 0.005 |
| >3 months | Anemia in pregnancy | -0.002 | -0.011, 0.006 | 0.597 | 0.004 |
| >3 months | Moderate/Severe Food Insecurity | 0.006 | -0.003, 0.014 | 0.182 | 0.004 |
| >3 months | Non diverse diet | -0.006 | -0.014, 0.002 | 0.172 | 0.004 |
| >3 months | ART initiation during pregnancy | 0.005 | -0.008, 0.018 | 0.446 | 0.006 |

**Supplementary Table 5:** Associations of characteristics of mother and children enrolled in Tunza Mwana study, Kenya with Stunting

| **Characteristics** | **Early stunting (≤3 months)** | | | | **Stunting at >3 months** | | | |
| --- | --- | --- | --- | --- | --- | --- | --- | --- |
|  | **RR (95% CI)** | ***P* value** | **aRR (95% CI)** | ***P* value** | **RR (95% CI)** | ***P* value** | **aRR (95% CI)** | ***P* value** |
| **Sociodemographic information​** |  |  |  |  |  |  |  |  |
| Age <25 years | 1.03 (0.62, 1.72) | 0.905 | 1.09 (0.66, 1.82) | 0.725 | 1.14 (0.87, 1.49) | 0.352 | 1.11 (0.85, 1.46) | 0.428 |
| Primary education or below | 1.5 (0.85, 2.65) | 0.163 | 1.38 (0.79, 2.43) | 0.261 | 1.34 (0.99, 1.81) | 0.060 | 1.28 (0.93, 1.76) | 0.132 |
| Unmarried^a^ | 0.88 (0.42, 1.84) | 0.732 | 0.7 (0.34, 1.45) | 0.342 | 1.03 (0.71, 1.48) | 0.883 | 0.9 (0.62, 1.32) | 0.603 |
| **Maternal anthropometry** |  |  |  |  |  |  |  |  |
| Shorter height (<Q1 157.3cm) | 1.89 (1.15, 3.11) | **0.012** | 2.33 (1.36, 3.98) | **0.002** | 1.39 (1.06, 1.83) | **0.017** | 1.36 (1.04, 1.79) | **0.026** |
| MUAC, cm | 0.96 (0.89, 1.04) | 0.376 | 0.97 (0.9, 1.05) | 0.505 | 0.94 (0.89, 0.98) | **0.007** | 0.95 (0.9, 1) | **0.029** |
| **Household factors** |  |  |  |  |  |  |  |  |
| Lower wealth quintile | 1.07 (0.91, 1.26) | 0.430 | 1.03 (0.87, 1.22) | 0.735 | 1.13 (1.03, 1.24) | **0.012** | 1.12 (1.02, 1.23) | **0.021** |
| Household crowding^c^ | 1.29 (0.63, 2.64) | 0.481 | 1.16 (0.5, 2.73) | 0.727 | 1.25 (0.86, 1.81) | 0.236 | 1.18 (0.8, 1.73) | 0.404 |
| No financial support the father | 0.83 (0.35, 1.96) | 0.672 | 0.68 (0.28, 1.63) | 0.387 | 1.18 (0.8, 1.72) | 0.403 | 1.02 (0.69, 1.49) | 0.935 |
| **Obstetric history and medical history** |  |  |  |  |  |  |  |  |
| Gestational age (weeks) | 0.78 (0.71, 0.86) | **0.000** | 0.78 (0.72, 0.86) | **0.000** | 0.97 (0.91, 1.03) | 0.352 | 0.97 (0.92, 1.03) | 0.365 |
| Preterm birth^d^ | 2.55 (1.47, 4.44) | **0.001** | 2.54 (1.43, 4.51) | **0.001** | 1.43 (1.01, 2.03) | **0.045** | 1.34 (0.94, 1.91) | 0.106 |
| Multiparous | 1.05 (0.56, 1.99) | 0.871 | 1.02 (0.47, 2.23) | 0.959 | 1.04 (0.74, 1.47) | 0.805 | 1.11 (0.76, 1.61) | 0.591 |
| Complicated pregnancy^e^ | 0.98 (0.57, 1.68) | 0.930 | 0.97 (0.57, 1.65) | 0.921 | 0.91 (0.67, 1.22) | 0.519 | 0.92 (0.69, 1.24) | 0.589 |
| No iron/folate supplementation | 1.15 (0.56, 2.36) | 0.714 | 0.99 (0.5, 1.98) | 0.987 | 0.89 (0.57, 1.38) | 0.599 | 0.88 (0.57, 1.36) | 0.564 |
| **Breastfeeding ​** |  |  |  |  |  |  |  |  |
| Exclusive breastfeeding <6 mo | 0.91 (0.54, 1.53) | 0.719 | 0.96 (0.58, 1.57) | 0.856 | 1.14 (0.87, 1.49) | 0.349 | 1.15 (0.88, 1.5) | 0.311 |
| **Psychosocial factors** |  |  |  |  |  |  |  |  |
| Perinatal depression/anxiety/IPV/ >2 ACEs^f^ | 1.09 (0.66, 1.8) | 0.742 | 1.01 (0.62, 1.64) | 0.979 | 1.07 (0.82, 1.4) | 0.616 | 1.01 (0.78, 1.32) | 0.917 |
| **Maternal nutrition and food insecurity** |  |  |  |  |  |  |  |  |
| Anemia (<11g/dL) | 0.87 (0.5, 1.51) | 0.613 | 0.83 (0.47, 1.44) | 0.502 | 1.07 (0.8, 1.42) | 0.661 | 1.06 (0.79, 1.40) | 0.708 |
| Moderate/Severe Food Insecurity | 1.21 (0.74, 1.99) | 0.455 | 1.16 (0.71, 1.89) | 0.557 | 1.00 (0.76, 1.31) | 0.981 | 1.03 (0.78, 1.35) | 0.851 |
| Minimum dietary diversity score | 0.93 (0.78, 1.11) | 0.449 | 0.93 (0.77, 1.13) | 0.477 | 0.93 (0.84, 1.02) | 0.130 | 0.94 (0.86, 1.04) | 0.256 |
| **Maternal HIV characteristics** |  |  |  |  |  |  |  |  |
| ART initiation during pregnancy | 0.76 (0.3, 1.88) | 0.546 | 0.76 (0.32, 1.80) | 0.527 | 0.77 (0.48, 1.22) | 0.26 | 0.72 (0.46, 1.14) | 0.159 |

*Reference categories:* ≥25 years (age); some secondary education or higher (education); married (marital status); ≥157.3 cm (height); higher wealth quintile; no crowding (≤3 persons/room); receiving financial support from father; ≥37 weeks’ gestation (preterm birth); no prior birth (parity); ≥6 months exclusive breastfeeding; food secure/mild food insecurity; ART initiation before conception.

^a^Unmarried includes divorced/separated/living together; ^b^Shorter height defined as <1st quartile 157.3 cm; ^c^Household crowding defined as >3 people/room in house; ^d^Preterm birth defined as <37 weeks of gestation; ^e^Complicated pregnancy includes infection, bleeding, high blood pressure, preeclampsia, or COVID during pregnancy, or hospitalized during pregnancy); ^f^Perinatal depression defined as PHQ9 score ≥ 5; anxiety GAD7 score ≥ 5;

RR or adjusted RR were obtained using Poisson regression with robust variance. Adjusted models included a priori covariates: maternal age (years), wealth index (0–4, highest to lowest quintile), gestational age (weeks), and infant sex. Models for age excluded continuous age; models for wealth excluded the wealth index; and models for gestational age and preterm birth excluded continuous gestational age.ACEs: Adverse Childhood Experience; ART: Antiretroviral Therapy; GAD-7: Generalized Anxiety Disorder 7-item; MUAC: Mid-upper Arm Circumference; PHQ-9: Patient Health Questionnaire-9; RR: Relative Risk

**Supplementary Table 6:** Associations of characteristics of mother and children enrolled in Tunza Mwana study, Kenya with Wasting

| **Characteristics** | **Early wasting (≤3 months)** | | | | **Wasting at >3 months** | | | |
| --- | --- | --- | --- | --- | --- | --- | --- | --- |
|  | **RR (95% CI)** | ***P* value** | **aRR (95% CI)** | ***P* value** | **RR (95% CI)** | ***P* value** | **aRR (95% CI)** | ***P* value** |
| **Sociodemographic information​** |  |  |  |  |  |  |  |  |
| Age <25 years | 1.00 (0.57, 1.75) | 0.994 | 1.03 (0.57, 1.85) | 0.927 | 0.52 (0.23, 1.19) | 0.124 | 0.45 (0.2, 1.05) | 0.065 |
| Primary education or below | 0.58 (0.34, 0.99) | **0.046** | 0.5 (0.27, 0.93) | **0.028** | 1.23 (0.58, 2.61) | 0.594 | 1.09 (0.47, 2.51) | 0.845 |
| Unmarried^a^ | 1.41 (0.73, 2.75) | 0.307 | 1.47 (0.71, 3.03) | 0.303 | 0.9 (0.33, 2.48) | 0.846 | 0.95 (0.34, 2.6) | 0.915 |
| **Maternal anthropometry** |  |  |  |  |  |  |  |  |
| Shorter height (<Q1 157.3cm) | 0.86 (0.45, 1.67) | 0.663 | 0.9 (0.46, 1.77) | 0.769 | 0.63 (0.25, 1.6) | 0.331 | 0.66 (0.25, 1.73) | 0.400 |
| MUAC, cm | 1.01 (0.93, 1.09) | 0.887 | 1 (0.93, 1.08) | 0.974 | 0.9 (0.76, 1.06) | 0.198 | 0.89 (0.75, 1.05) | 0.175 |
| **Household factors** |  |  |  |  |  |  |  |  |
| Lower wealth quintile | 0.97 (0.79, 1.190) | 0.762 | 0.96 (0.79, 1.18) | 0.700 | 1.15 (0.9, 1.46) | 0.274 | 1.1 (0.86, 1.41) | 0.427 |
| Household crowding^c^ | 0.39 (0.10, 1.53) | 0.175 | 0.38 (0.10, 1.50) | 0.169 | 1.73 (0.71, 4.25) | 0.230 | 1.48 (0.61, 3.55) | 0.385 |
| No financial support the father | 1.69 (0.85, 3.35) | 0.133 | 1.77 (0.86, 3.66) | 0.122 | 1.25 (0.46, 3.39) | 0.662 | 1.1 (0.39, 3.1) | 0.849 |
| **Obstetric history and medical history** |  |  |  |  |  |  |  |  |
| Gestational age (weeks) | 0.92 (0.8, 1.07) | 0.275 | 0.92 (0.79, 1.07) | 0.278 | 1.01 (0.87, 1.18) | 0.903 | 1.02 (0.89, 1.17) | 0.747 |
| Preterm birth^d^ | 1.46 (0.67, 3.18) | 0.336 | 1.51 (0.68, 3.36) | 0.308 | 1.10 (0.35, 3.42) | 0.872 | 0.99 (0.33, 2.95) | 0.985 |
| Multiparous | 0.88 (0.46, 1.68) | 0.695 | 0.78 (0.38, 1.62) | 0.508 | 0.79 (0.35, 1.77) | 0.564 | 0.46 (0.16, 1.3) | 0.143 |
| Complicated pregnancy^e^ | 1.10 (0.62, 1.95) | 0.749 | 1.08 (0.61, 1.94) | 0.784 | 1.16 (0.56, 2.4) | 0.697 | 1.23 (0.6, 2.5) | 0.567 |
| No iron/folate supplementation | 0.92 (0.39, 2.19) | 0.850 | 0.87 (0.36, 2.1) | 0.764 | 0.54 (0.14, 2.2) | 0.395 | 0.52 (0.13, 2.01) | 0.343 |
| **Breastfeeding ​** |  |  |  |  |  |  |  |  |
| Exclusive breastfeeding <6 mo | 1.14 (0.65, 1.98) | 0.655 | 1.15 (0.66, 1.98) | 0.628 | 1.23 (0.6, 2.55) | 0.570 | 1.23 (0.6, 2.52) | 0.578 |
| **Psychosocial factors** |  |  |  |  |  |  |  |  |
| Perinatal depression/anxiety/IPV/ >2 ACEs^f^ | 0.82 (0.48, 1.42) | 0.488 | 0.82 (0.48, 1.41) | 0.476 | 0.7 (0.35, 1.41) | 0.319 | 0.64 (0.32, 1.25) | 0.191 |
| **Maternal nutrition and food insecurity** |  |  |  |  |  |  |  |  |
| Anemia (<11g/dL) | 0.74 (0.39, 1.41) | 0.356 | 0.74 (0.39, 1.4) | 0.353 | 1.67 (0.81, 3.42) | 0.166 | 1.62 (0.79, 3.31) | 0.187 |
| Moderate/Severe Food Insecurity | 0.70 (0.40, 1.26) | 0.237 | 0.68 (0.38, 1.2) | 0.182 | 0.64 (0.3, 1.35) | 0.238 | 0.72 (0.34, 1.54) | 0.397 |
| Minimum dietary diversity score | 1.00 (0.78, 1.27) | 0.990 | 0.99 (0.78, 1.26) | 0.945 | 0.79 (0.62, 1.01) | 0.062 | 0.81 (0.63, 1.03) | 0.082 |
| **Maternal HIV characteristics** |  |  |  |  |  |  |  |  |
| ART initiation during pregnancy | 1 (0.43, 2.34) | 0.995 | 1.08 (0.47, 2.48) | 0.852 | 0.17 (0.02, 1.21) | 0.076 | 0.15 (0.02, 1.04) | 0.055 |

*Reference categories:* ≥25 years (age); some secondary education or higher (education); married (marital status); ≥157.3 cm (height); higher wealth quintile; no crowding (≤3 persons/room); receiving financial support from father; ≥37 weeks’ gestation (preterm birth); no prior birth (parity); ≥6 months exclusive breastfeeding; food secure/mild food insecurity; ART initiation before conception.

^a^Unmarried includes divorced/separated/living together; ^b^Shorter height defined as <1st quartile 157.3 cm; ^c^Household crowding defined as >3 people/room in house; ^d^Preterm birth defined as <37 weeks of gestation; ^e^Complicated pregnancy includes infection, bleeding, high blood pressure, preeclampsia, or COVID during pregnancy, or hospitalized during pregnancy); ^f^Perinatal depression defined as PHQ9 score ≥ 5; anxiety GAD7 score ≥ 5;

RR or adjusted RR were obtained using Poisson regression with robust variance. Adjusted models included a priori covariates: maternal age (years), wealth index (0–4, highest to lowest quintile), gestational age (weeks), and infant sex. Models for age excluded continuous age; models for wealth excluded the wealth index; and models for gestational age and preterm birth excluded continuous gestational age.ACEs: Adverse Childhood Experience; ART: Antiretroviral Therapy; GAD-7: Generalized Anxiety Disorder 7-item; MUAC: Mid-upper Arm Circumference; PHQ-9: Patient Health Questionnaire-9; RR: Relative Risk
