# Supplementary figures and images for: Maternal Health and Sociodemographic Characteristics Influence Infant Growth to 24 Months in the Tunza Mwana Cohort: A Prospective Cohort Study
Source: Matern Child Nutr. 2026 May 14;22(3):e70195. doi: 10.1111/mcn.70195 (PMC13176631; doi:10.1111/mcn.70195)

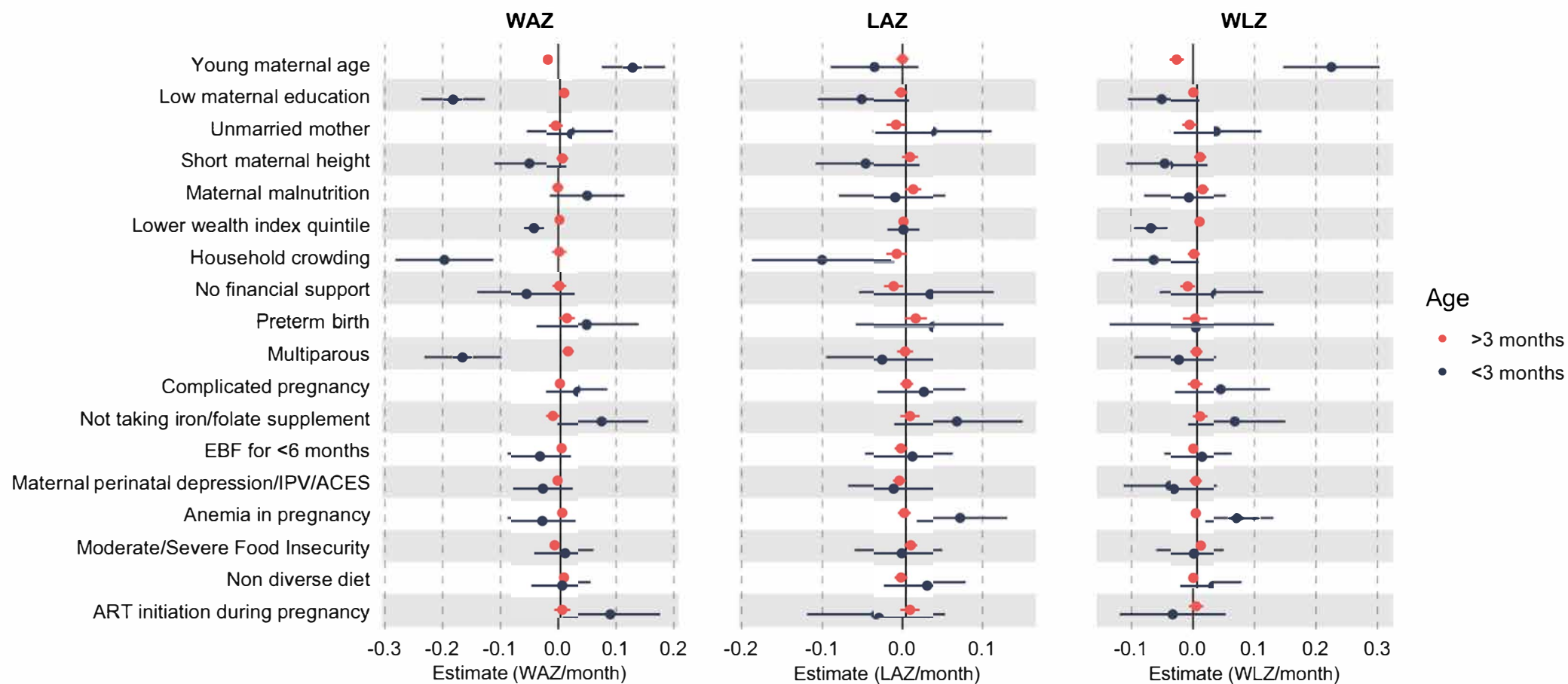

Supplement: Supplementary file 2 — Supporting File 2 [file MCN-22-e70195-s001.pdf]
